# Effect of patient position on the lordosis and scoliosis of patients with degenerative lumbar scoliosis

**DOI:** 10.1097/MD.0000000000007648

**Published:** 2017-08-11

**Authors:** Han Fei, Wei-shi Li, Zhuo-ran Sun, Shuai Jiang, Zhong-qiang Chen

**Affiliations:** Department of Orthopaedics, Peking University Third Hospital, Beijing, China.

**Keywords:** degenerative lumbar scoliosis, intraoperative prone position, lumbar lordosis, scoliosis, surgical plan

## Abstract

This study aimed to analyze the effect of patient positions on the lordosis and scoliosis of patients with degenerative lumbar scoliosis (DLS).

Seventy-seven patients with DLS were retrospectively analyzed. We measured lordosis and Cobb's angle on preoperative upright x-rays and magnetic resonance imagings in supine position. The lordosis and scoliosis of surgical segments in intraoperative prone position were measured on intraoperative radiographs of 20 patients to compare with that in standing position. Paired *t* tests were performed to investigate the parameters of the sample.

From standing to supine position the whole lordosis increased (29.2 ± 15.7 degree vs. 34.9 ± 11.2 degree), and the whole scoliosis decreased (24.3 ± 11.8 degree vs. 19.0 ± 10.5 degree); 53 of 77 (68.8%) cases had increased lordosis, and 67 of 77 (87%) cases had decreased scoliosis. The lordosis of surgical segments in standing position had no difference with that in intraoprerative prone position. But in changing from supine/standing position to intraoprerative prone position, the scoliosis of surgical segments decreased (14.7 ± 9.4 degree vs. 11.4 ± 7.0 degree; 19.0 ± 11.8 degree vs. 11.4 ± 7.0 degree, respectively), and 18 of 20 (90%) cases had decreased scoliosis in intraoperative prone position than that in standing position.

Compared with standing position in DLS patients, supine position increased lordosis and reduced scoliosis, and intraoperative prone position reduced scoliosis significantly. When evaluating the severity of DLS and making preoperative surgical plans, lumbar lordosis in supine position should also be evaluated in addition to upright x-ray, and the effects of different positions should be taken into consideration to reduce deviation.

## 1 Introduction

Scoliosis and lower lumbar lordosis (LL) are commonly seen in patients with degenerative lumbar scoliosis (DLS).^[1,2]^ As we all know, on one hand, that correction of scoliosis is an important part of DLS treatment, on the other hand, correction of LL is an essential prerequisite to restoration of sagittal balance, which is also associated with clinical outcomes.^[3,4]^

An x-ray of the whole spine in standing position is often used to evaluate the scoliosis for surgical decisions, and several methods for correction of LL have been proposed, especially the equation using pelvic incidence (PI) for guiding the extent of LL correction (LL = PI ± 9 degree)^[5,6]^; these methods were obtained from patients in upright position. Moreover, preoperative x-rays are also used for determining the location of surgical incisions and adjusting the direction of pedicle screws. However, the spinal alignment in supine position when undergoing computed tomography or magnetic resonance imaging (MRI) and the prone position during operations may be different from that in standing position, which results in deviation in surgical plans.

Previous studies have investigated the commonly adopted postures and their effects on the lumbar spine,^[7–13]^ but none of them focused on DLS patients. In this study, we investigated the lordosis and scoliosis of different patient positions in DLS, and provide some useful information for planning surgery.

## 2 Materials and methods

The study protocol was approved by the institutional ethics committee at our institution; subjects received no extra treatment during the study process. Data pertaining to 77 patients (older than 40 years) with DLS who underwent long-segment fixation (at least 4 vertebra) at our hospital during 2009 to 2015 were retrospectively analyzed. The cases were selected randomly, and included once it met the inclusion criteria. All patients were diagnosed with DLS according to clinical manifestation and radiological examination by a group of experienced experts, mainly complaining about low back pain and claudication. The exclusion criteria were: patients with spinal tumor; adolescent idiopathic scoliosis (AIS); isthmic spondylolisthesis; history of pelvic fracture, fixation of spine or lower extremities; and scoliosis caused by a forced posture. Twenty of them were investigated during operation, using a C-arm machine to take intraoperative x-rays.

**Figure 1:**
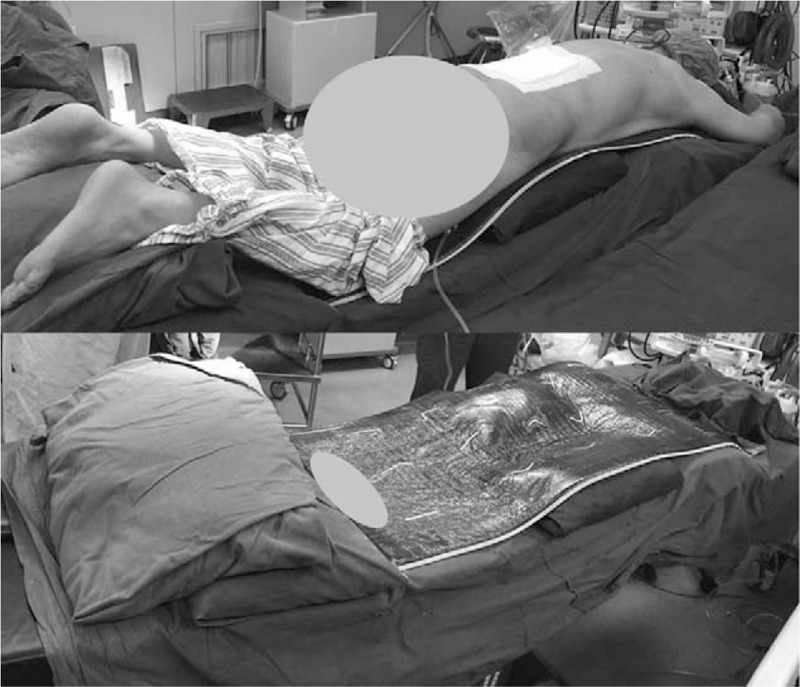
View of a patient positioned prone in a standard manner and the operating table. Two chest pads and 2 iliac pads were placed to allow the abdomen to hang free. The knees and shins were also placed on pads. The operating table was adjusted to make the hip and the knees in slight flexion.

The parameters in Table 1 were measured using the Picture Archiving and Communication System (PACS system, GE) and surgimap spine (Nemaris). We measured LL and Cobb angle on preoperative standard anteroposterior and lateral radiographs (standing position, with full extension of hips and knees, elbow flexion and hands on the clavicle, including bilateral femoral heads). Preoperative MRI was also available, through which LL measured on MRI in supine position (MRLL) and Cobb's angle measured on MRI in supine position (MRCobb) were obtained. The 20 patients investigated were positioned in a standard manner on an operating table. The operating tables were of the same type; 2 chest pads and 2 iliac pads were placed to allow the abdomen to hang free. The knees and shins were also placed on pads of the same height. The operating table was adjusted to make the hips and the knees in slight flexion (Fig. 1). The intraoperative C-arm x-rays of the 20 patients were taken during the process of pedicle screw placement before the completion of internal fixation. As the intraoperative images of the whole lumbar spine were not always recorded, the pre- and intraoperative parameters of the surgical segments were analyzed. If the measurements were restricted by the projection range of C-arm, section images were spliced to obtain the total angle. The osteophyma was not added in the calculation of measurement. All measurements were done twice by 1 experienced observer and a mean value was adopted.

**Table 1:**
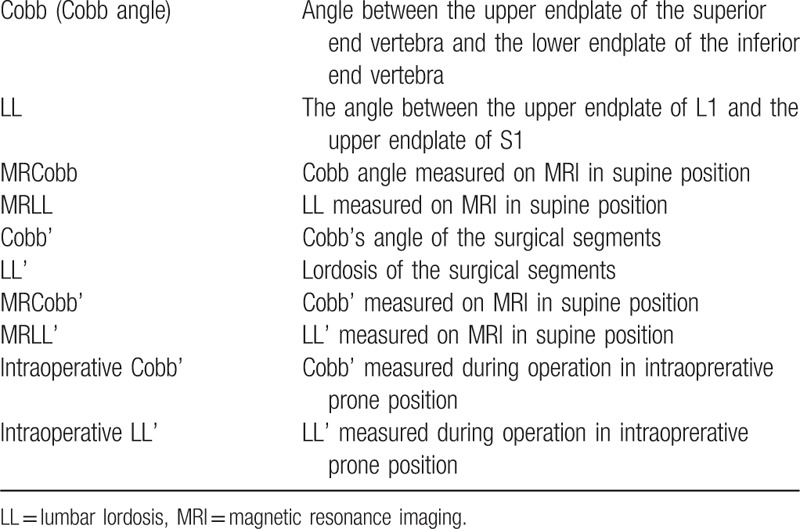
Radiographic measurements.

A paired *t* test was used to compare the parameters of the sample. All data analyses were performed using SPSS 22.0 statistical software (SPSS Inc, Chicago, IL). The statistical level of significance was set at 0.05.

## 3 Results

Of the patients in this study, 59 (77%) were female and 18 (23%) were male, and the mean age was 63.0 ± 6.8 years (40∼76 years). The mean Cobb angle was 24.3 ± 11.7 degree (10.0∼52.3 degree), and the mean LL was 29.2 ± 15.7 degree (−19.7∼65.4 degree).

Parameters reflecting lordosis and scoliosis from standing position to supine position are listed on Table 2. A paired *t* test showed that the mean MRLL was higher than LL (34.9 ± 11.2 degree vs. 29.2 ± 15.7 degree, *P* < .001), suggesting that the whole lordosis increased from standing to supine position, and 52 of 77 cases (68.8%) had higher MRLL than LL. And MRCobb was lower than Cobb (19.0 ± 10.5 degree vs. 24.3 ± 11.8 degree, *P* < .001), suggesting that the whole scoliosis decreased from standing to supine position. Sixty-seven of 77 cases (87%) had lower MRCobb than Cobb.

**Table 2:**
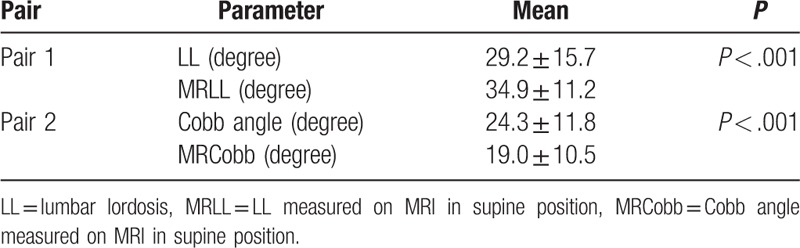
Comparison of LL in upright position and MRLL in supine position (n = 77).

Preoperative parameters of surgical segments in upright/supine position and intraoprerative parameters of surgical segments in intraoprerative prone position are listed on Table 3. Compared with the intraoprerative LL’ in intraoprerative prone position (24.6 ± 10.8 degree), the LL’ in standing position (23.5 ± 12.7 degree) had no difference (*P* > .05).

**Table 3:**
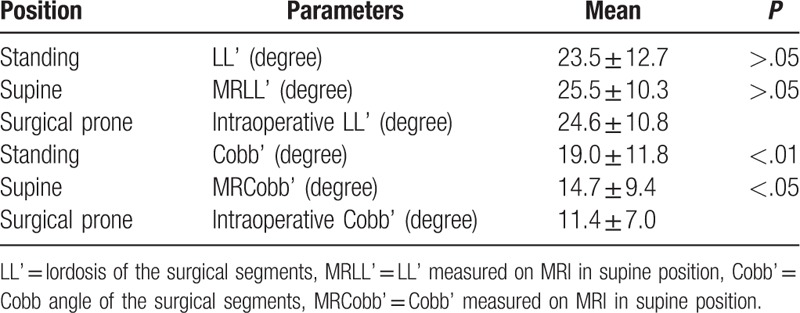
Comparison of preoperative parameters in upright/supine position and intraoprerative parameters in intraoprerative prone position (surgical segments, n = 20, paired *t* test).

**Figure 2:**
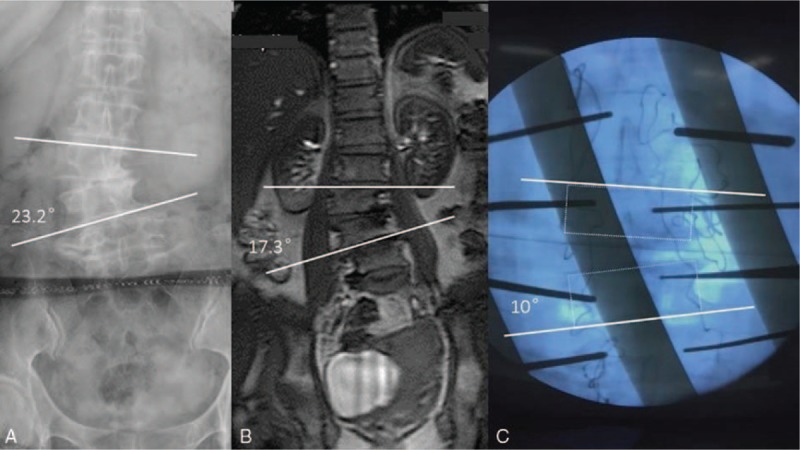
Radiographic example of a patient (female, 63 years) with degenerative lumbar scoliosis in 3 positions: (A) preoperative upright posteroanterior radiograph shows 23.2 degree of scoliosis; (B) in preoperative MR in supine position the Cobb angle reduced to 17.3 degree. (C) Positioned prone during the process of pedicle screw insertion from L2 to L5, the Cobb angle further reduced to 10 degree.

However, in changing from supine position to intraoprerative prone position, there were statistically significant differences between MRCobb’ and intraoprerative Cobb’ (14.7 ± 9.4 degree vs. 11.4 ± 7.0 degree, *P* < .05), suggesting the scoliosis of the surgical segments decreased (Fig. 2), and the difference between Cobb’ in standing position and intraoprerative Cobb’ was also significant (19.0 ± 11.8 degree vs. 11.4 ± 7.0 degree, *P* < .01), suggesting the scoliosis of the surgical segments significantly decreased (Fig. 2). Of the 20 cases observed during operation, 15 (75%) cases had lower intraoperative Cobb’ than Cobb’ in supine position, and 18 (90%) cases had lower intraoperative Cobb’ than Cobb’ in standing position.

## 4 Discussion

In DLS corrective surgery, not only scoliosis but also lordosis should be appropriately corrected. Presently surgeons restore the lordosis by bending the rod according to the surgeon's own experience or the formula (e.g., LL = PI ± 9 degree), and recently we have found that PI-LL = 12∼28 degree may be more suitable for DLS patients. However, these formulas did not take the influence of patient position into consideration. The corrective plans refer to the preoperative x-rays in standing position, and during regular posterior operation patients are often placed prone rather than standing or supine, and the application of muscle relaxant, muscle incision, and release of soft tissue may relieve the scoliosis. So the intraoperative lordosis and scoliosis may be different, resulting in imprecise operative plan. And if the intraoperative scoliosis decreases, the necessity of osteotomy may also decrease. Preoperative x-rays are also referred in controlling the direction of pedicle screw placement, and the difference of curvature caused by intraoperative position may bring error to the direction of pedicle screws. In addition, some patients with severe pain and muscle weakness are not able to keep a standing posture, so images in supine position may be alternative. To indentify the effects of patient positions, reduce such deviations, and make the corrective surgery more precise and safe, the differences between 3 positions were analyzed in this study.

This study investigated the change of intraoperative LL, and found there was no statistical difference in intraoprerative lordosis of surgical segments compared with that in standing position, which was in accordance with the previous literature. Peterson et al^[14]^ and Stephens et al^[15]^ reported that for patients with lumbar spine diseases and asymptomatic volunteers, prone position on the Jackson table reproduces the physiologic lordosis. Tan et al^[16]^ also found no significant difference in LL between the standing and chest rolls positions. Benfanti et al^[17]^ found that in anesthetized lumbar fusion patients, standardized positioned on the Wilson frame preserved 95% of preoperative standing lordosis. Marsicano et al^[18]^ reported that intraoperative total LL in Orthopedic Systems Incorporated frame in AIS patients was maintained from the preoperative state. Harimaya et al^[19]^ found that adult spinal deformity patients with preoperative hypolordosis positioned prone during reconstructive surgery had an increased lordosis compared with their preoperative radiographs, whereas the lordosis in those with substantial preoperative lordosis remained unchanged, and they suggested the preoperative supine radiograph helps predict the intraoperative LL needed. This suggests that the lordosis during operation is approximately equal to that in standing position, and add to the theoretical evidence for making surgical plans and adjusting the direction of pedicle screw based on the x-ray in standing position. But this study analyzed the lordosis of instrumented segments; further studies about the change of whole lordosis are needed.

However, in this study, the lordosis increased from standing to supine position, which was different from the previous literature. Andreasen et al^[8]^ showed that LL of patients with radicular pain in upright position can be reproduced by positioning the patient supine with straightened lower extremities, but Wood et al^[12]^ found that asymptomatic individuals and patients with low back pain demonstrated small increases in LL when standing versus supine; Mauch et al^[11]^ also found the lordosis of 35 athletes increased by 14% from supine to true standing position. Such difference may be derived from the subjects investigated; in this study, DLS patients were studied, there were sagittal deformities accompanied by coronal deformities, with lower LL and more forward sagittal spinopelvic decompensation,^[1,2]^ whereas in supine position the weight bear disappears, which reduced the scoliosis and vertebrae rotation, and therefore the sagittal deformities, then LL was restored partially. Moreover, the supine images were obtained from MRI, during which the body was clinging to the MRI machine and kept straight, with the lower limbs fully extended and the pelvis rotated forwardly, which contributed to the increase of LL. In addition, the pain-related muscle contraction (voluntary or involuntary) could lead to decrease of LL in standing position,^[20]^ whereas in supine position, the muscle cramps were reduced and the LL was restored. This information is helpful for patients unable to take standing x-rays because of severe pain and muscle weakness, and when using supine images for a substitute, the change of LL should be noticed.

In terms of lumbar scoliosis, previous studies have found a 9∼10 degree decrease of scoliosis from standing to supine position in AIS,^[21–23]^ but this change of scoliosis in DLS remains unclear. In this study, the scoliosis of DLS patients decreased from standing to supine position (24.3 ± 11.8 degree vs. 19.0 ± 10.5 degree), indicating that the scoliosis in supine position is not so serious as that in upright position. Absence of weight bear and muscle relaxants may be the reason of this reduction, and this difference should be taken into consideration when evaluating the severity of DLS.

To the best of our knowledge, the intraoperative change of DLS has not been reported. This study observed a significant decrease of surgical segments scoliosis from standing to surgical prone position (19.0 ± 11.8 degree vs. 11.4 ± 7.0 degree). Two major factors may be responsible. First, in positioning from upright to prone, the weight bear decreased along with the decrease of the vertical pressure, and so was the deformities induced by stress. Second, during surgery, the muscle tension was eliminated under general anesthesia with muscle relaxant, muscle dissection, and adhesion release. Thus, the muscular imbalance was partly corrected, resulting in a smaller scoliosis. This information is important for surgeons because if this difference is neglected, there will be lots of deviation in the preoperative plan for scoliosis correction based on upright x-rays. This factor should also be taken into account when determining the angle of pedicle screw insertion using preoperative x-rays because the change of scoliosis will affect the appropriate angle of pedicle screw, especially in axial plane. With fully consideration of the effects of patient positions and timely adjustment, the insertion of pedicle screws will be more precise and the risk of complications (pedicle fracture/spinal cord injury) may be reduced.

This study has some limitations. As a retrospective study, there may still be differences between surgeons in positioning the patients despite the height and locations of the pads remained the same. And there was no preoperative supine x-ray to measure the Cobb angle, so we had to use preoperative MRI, although the measurement on MRI has been proved to be reliable and of little variation than that on x-ray,^[12,21–24]^ there may be deviation when comparing measurements on MRI with that on x-ray. Meanwhile, as the projection range of C-arm was limited, segment images were spliced to obtain the total angle, which may also produce deviations. Nevertheless, this study provides useful information about effect of patient position on lordosis and scoliosis of DLS patients, which has reference value for making corrective plans and ensuring surgical safety.

## 5 Conclusion

In positioning the DLS patients from standing to supine position, the LL increased, and the scoliosis decreased. After changing from standing to surgical prone position, the surgical segments had a significant decrease of scoliosis, and the lordosis remained roughly the same. In preoperative evaluation of DLS, making corrective plans, and adjusting the direction of pedicle screw, this difference should be taken into consideration rather than using the preoperative upright x-ray directly and simply.
